# Patients with Multiple Myeloma Develop SOX2-Specific Autoantibodies after Allogeneic Stem Cell Transplantation

**DOI:** 10.1155/2011/302145

**Published:** 2011-11-15

**Authors:** Sebastian Kobold, Sinje Tams, Tim Luetkens, Yanran Cao, Orhan Sezer, Britta Marlen Bartels, Henrike Reinhard, Julia Templin, Katrin Bartels, York Hildebrandt, Nesrine Lajmi, Andreas Marx, Friedrich Haag, Carsten Bokemeyer, Nicolaus Kröger, Djordje Atanackovic

**Affiliations:** ^1^Department of Internal Medicine II, Oncology/Hematology/Bone Marrow Transplantation with the Section Pneumology, University Medical Center Hamburg-Eppendorf, 20246 Hamburg, Germany; ^2^University Cancer Center Hamburg (Hubertus Wald Tumorzentrum), University Medical Center Hamburg-Eppendorf, 20246 Hamburg, Germany; ^3^Division of Clinical Pharmacology, Department of Internal Medicine, Ludwig-Maximilian University, 80336 Munich, Germany; ^4^Department of Stem Cell Transplantation, University Medical Center Hamburg-Eppendorf, 20246 Hamburg, Germany; ^5^Institute for Pathology, University Medical Center Hamburg-Eppendorf, 20246 Hamburg, Germany; ^6^Institute for Immunology, University Medical Center Hamburg-Eppendorf, 20246 Hamburg, Germany

## Abstract

The occurrence of SOX2-specific autoantibodies seems to be associated with an improved prognosis in patients with monoclonal gammopathy of undetermined significance (MGUS). However, it is unclear if SOX2-specific antibodies also develop in established multiple myeloma (MM). Screening 1094 peripheral blood (PB) sera from 196 MM patients and 100 PB sera from healthy donors, we detected SOX2-specific autoantibodies in 7.7% and 2.0% of patients and donors, respectively. We identified SOX2_211–230_ as an immunodominant antibody-epitope within the full protein sequence. SOX2 antigen was expressed in most healthy tissues and its expression did not correlate with the number of BM-resident plasma cells. Accordingly, anti-SOX2 immunity was not related to SOX2 expression levels or tumor burden in the patients' BM. The only clinical factor predicting the development of anti-SOX2 immunity was application of allogeneic stem cell transplantation (alloSCT). Anti-SOX2 antibodies occurred more frequently in patients who had received alloSCT (*n* = 74). Moreover, most SOX2-seropositive patients had only developed antibodies after alloSCT. This finding indicates that alloSCT is able to break tolerance towards this commonly expressed antigen. The questions whether SOX2-specific autoantibodies merely represent an epiphenomenon, are related to graft-versus-host effects or participate in the immune control of myeloma needs to be answered in prospective studies.

## 1. Introduction

SRY-related HMG box (SOX) is a superfamily of transcription factors involved in embryonic development and stem cell function [1]. Cancer cells share pathways regulating pluripotency with embryonal stem cells [2], and some of the transcription factors involved, including SOX2, have indeed been identified as lineage survival oncogenes in epithelial cancers [3]. The impact of SOX2-specific immunity on the patient's prognosis has been investigated in single solid tumors [2]. However, the exact biological role of cancer-related SOX2-specific antibody and/or T cell responses has remained unclear. Accordingly, some studies have suggested an association with an improved prognosis while others have found no association with the patients' outcome or have even described a negative impact on the course of the disease [4–6].

Monoclonal gammopathy of undetermined significance (MGUS) is a premalignancy converting to symptomatic multiple myeloma (MM) at a rate of 1-2% of patients per year [7]. The prevalence of SOX2-specific antibodies in MGUS patients has been linked to a decreased risk of progression to MM [8]. However, SOX2 is expressed not only in MGUS but also in symptomatic MM [6], and it has remained unclear if and under which clinical conditions autoantibodies against SOX2 also occur in established MM. Moreover, allogeneic stem cell transplantation (alloSCT) has been suggested to break tolerance towards different tumor antigens in MM resulting in a clinically relevant graft-versus-myeloma (GvM) effect. The question is still open, however, if alloSCT also influences the development of anti-SOX2 immunity in patients with established MM [9, 10]. To address these issues, we performed a longitudinal analysis of SOX-specific antibodies in patients with established MM.

## 2. Material and Methods

### 2.1. Patients

Patients were admitted for diagnostic purposes and/or treatment to the University Medical Center Hamburg-Eppendorf. Repeated blood samples were obtained during routine diagnostic procedures and all participants provided informed consent prior to sample collection. A total of 1094 peripheral blood (PB) plasma samples and 25 bone marrow (BM) samples were collected from 194 consecutive MM patients. In addition, 100 PB sera and 10 BM samples were collected from healthy donors. Samples were collected as previously described [11]. This study was conducted in accordance with the declaration of Helsinki. The protocol had received approval by the local ethics committee (decision number OB-038/06).

### 2.2. Myeloma Cell Lines

Cell lines U266, RPMI 8266, LP1, OPM2, NCIH929, MOPL8, KMS12BM, IM9, and EJM were obtained from the German Collection of Microorganisms and Cell cultures (DSMZ, Braunschweig, Germany). Cell line SK 007 was provided by the Ludwig Institute for Cancer Research (LICR), New York branch. Cell lines were maintained in RPMI 1640 and 10% fetal calf serum [12].

### 2.3. Proteins and Peptides

Full-length SOX2 protein and control protein glutathione *S*-transferase (GST) were expressed in a wheat germ system (Abnova, Taipei, Taiwan). Recombinant influenza nucleoprotein (FLU) produced in *E. coli* was obtained from Imgenex (San Diego, Calif) and tetanus toxoid (TT) was provided by Chiron Behring (Marburg, Germany). Control protein for FLU and TT antibody detection was GST expressed in *E. coli* (Cell Systems, St Katharinen, Germany). 20 mer SOX2 peptides (*n* = 31) spanning the entire SOX2 sequence consisting of 317 amino acids were obtained from Iris Biotech (Marktredwitz, Germany).

### 2.4. Enzyme-Linked Immunosorbent Assay (ELISA)

96-well plates were coated over night at 4°C with recombinant protein or peptides diluted in PBS at a final concentration of 1 *μ*g/mL, if not otherwise specified. Plates were blocked with PBS containing 3% milk powder for two hours at room temperature (RT). Sera were diluted 1 : 100 in 5% milk powder in PBS (MPBS) and incubated for two hours at RT. A secondary alkaline phosphatase-conjugated anti-human IgG antibody (Southern Biotech, Birmingham, Ala) diluted 1 : 3000 in MPBS was applied for one hour at RT. Detection reagent para-nitrophenylphosphate (PNPP; Southern Biotech) was added to the plates, and the phosphatase reaction took place at RT for 30 minutes, before reaction arrest with 3 N NaOH. Specific absorption was measured at 405 nm using a Sunrise ELISA reader (Tecan, Crailsheim, Germany).

In the screening part of the study, a sample was considered antibody positive if the OD measured was higher than the mean OD of 100 samples from healthy donors + 3 SD. In addition, the OD was required to exceed the autologous background signal measured with control protein GST by at least 50%. In the titration part of the study, serial serum dilutions were performed for antibody-positive samples, and results obtained with GST protein were used as reference values. For calculation of titers, regression analyses were performed for the linear segment of the serum titration curves for the patient sample and pooled sera of five representative healthy donors. Titers were defined mathematically as the dilution at the intersection of both regression lines.

### 2.5. Real-Time PCR

Extraction of total RNA was performed using the RNeasy Mini Kit (Qiagen, Hilden, Germany). Reverse transcription and quantitative PCR were performed as previously described [10]. The primer sequences for SOX2 were as follows: forward 5′-GCA CAT GAA CGG CTG GAG CAA CG-3′, reverse 5′-TGC TGC GAG TAG GAC ATG CTG TAG-3′. Samples were analyzed using a LightCycler system (Roche Diagnostics, Risch, Switzerland), and relative quantification was carried out by normalization against GAPDH RNA.

### 2.6. Western Blot

Protein lysates were prepared using standard lysis buffer containing a protease inhibitor cocktail (Sigma-Aldrich, Hamburg, Germany) and were subsequently denaturated for 10 min at 70°C. Samples of lysates or recombinant protein containing 500 *μ*g and 300 *μ*g of total protein, respectively, were separated using 4–12% Bis-Tris SDS-PAGE gels (Invitrogen, Carlsbad, Calif) under reducing conditions. Proteins were blotted on Hybond-ECL nitrocellulose membranes (Amersham Biosciences, Piscataway, NJ), blocked overnight at 4°C with Top-Block (Fluka, Buchs, Switzerland). Human sera were applied at a dilution ranging between 1 : 500 and 1 : 2000. An HRP-conjugated anti-human IgG-Fc*γ* antibody (Sigma Aldrich) was used as secondary antibody at a dilution of 1 : 5000. *β*-Actin (Santa Cruz) served as loading control.

### 2.7. Flow Cytometry

For the analysis of cytoplasmatic SOX2 protein expression, myeloma cell lines or bone marrow mononuclear cells were first stained using a CD138-FITC monoclonal antibody (clone B-A38, BD Biosciences). Next, cells were fixed using FACS Lysing Solution (BD Biosciences) and were permeabilized using Permeabilizing Solution (BD Biosciences). Cytoplasmic staining was performed applying a PE-conjugated SOX2 antibody (clone IC2018P, R&D, Abington, England) or an appropriate isotype control. Samples were analyzed using a FACSCalibur cytometer (BD Biosciences) and FlowJo software (Tree Star, Ashland, Ore).

### 2.8. Epitope Prediction

Web-based prediction of potential SOX2 antibody epitopes (http://www.cbs.dtu.dk/services/BepiPred) was performed using the method published by Larsen et al. [13].

### 2.9. Statistical Analysis

Statistical analyses were performed using GraphPad software. The Mann-Whitney *U* test was used to calculate differences between different patient cohorts. Analysis of covariance was used to assess correlations between plasma cell count, SOX2 antibody titers, and SOX2 expression. Correlations between clinicopathological variables and occurrence of SOX2 antibodies were done by Pearson's *χ* ^2^ test. Differences were regarded significant if *P* < 0.05.

## 3. Results

### 3.1. SOX2 Is Expressed in Various Healthy Tissues and Malignant Myeloma Cells

**Figure 1:**
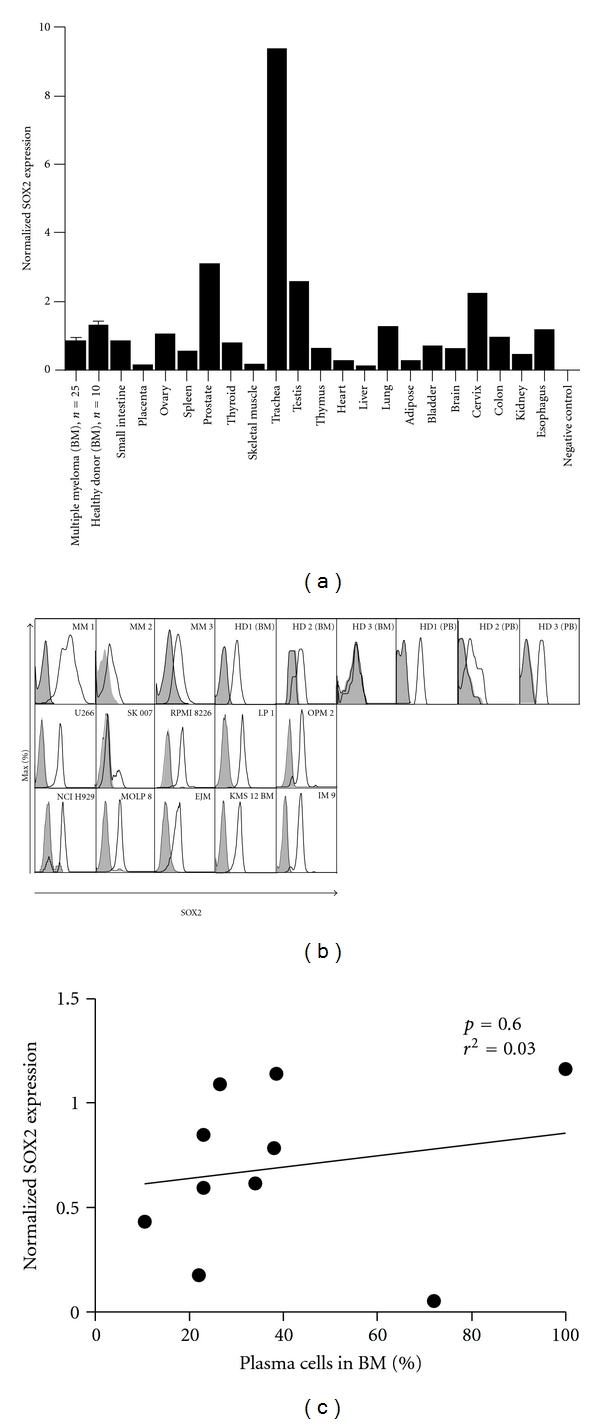
(a) RT-PCR analysis of SOX2 expression normalized to GAPDH in human tissues. BM from MM patients (*n* = 25), healthy donors (*n* = 15), myeloma cell lines (*n* = 10), and 20 human tissues (*n* = 1) was screened for SOX2 expression. Aqua dest. and non-reverse-transcribed mRNA were used as negative controls. 20 organs were tested for the presence of contaminating DNA. The resulting copy numbers (reverse-transcriptase-free) were normalized to GAPDH copy number of the respective tissue (cDNA). The mean value of all reverse-transcriptase-free results was calculated and included as the reverse-transcriptase-free (RT-free) condition. (b) FACS analysis of three MM patients' BM, three BM of healthy donors, and three peripheral blood samples of healthy donors for SOX2 expression in gated CD138+ plasma cells. One BM sample (3) was found negative for SO2 protein expression. SOX2 expression was also found in 10 different myeloma cell lines. Isotype antibodies served as negative control for SOX2 expression. (c) Correlation analysis of SOX2 expression and percentage of plasma cells in the BM of MM patients. No significant association between SOX2 expression and the amount of plasma cells was found (*P* = 0.6018, *r* ^2^ = 0.03556). HD: healthy donor; MM: multiple myeloma; BM: bone marrow; PB: peripheral blood.

SOX2 has been reported to be overexpressed in malignancies [14–16], and overexpression of SOX2 has been associated with immunity towards autologous antigens in cancer patients [17]. Therefore, we first addressed the expression of SOX2 in the BM of MM patients compared to other tissues. To this end, we screened a wide variety of normal tissues including 10 BM samples from healthy donors as well as BM samples from 25 MM patients for SOX2 expression by real-time PCR (Figure 1(a)).

We found SOX2 RNA to be ubiquitously expressed in all tissues analyzed. SOX2 is an intronless gene, and, therefore, this expression could also represent an artifact due to the presence of genomic DNA within the samples. However, we could rule out this possibility by showing that no SOX2 expression was detectable when the PCR was performed with non-reverse-transcribed RNA samples (Figure 1(a)). Importantly, we did not detect any significant differences in BM expression of SOX2 between myeloma patients and healthy donors (Figure 1(a)). To prove the presence of SOX2 on the protein level, we have performed flow cytometry analysis of myeloma cell lines and of plasma cells of healthy donors, all of which we found positive for SOX2 by RT-PCR. Importantly, all of the myeloma cell lines, all peripheral, and one of two bone marrow-derived plasma cell samples were also found positive for SOX2 on the protein level (Figure 1(b)). These data demonstrate a strong correlation between expression on the RNA level and protein expression as indicated by flow cytometry. As suggested by the comparable expression of SOX2 in normal and malignant plasma cells and its broad expression in different healthy tissues, copy numbers of SOX2 RNA as measured by quantitative PCR did not correlate with the percentage of myeloma cells within the BM of our MM patients (Figure 1(c)).

### 3.2. Antibodies against SOX2 Recognize the Natural Protein and Occur More Frequently in MM Patients Than in Healthy Donors

**Figure 2:**
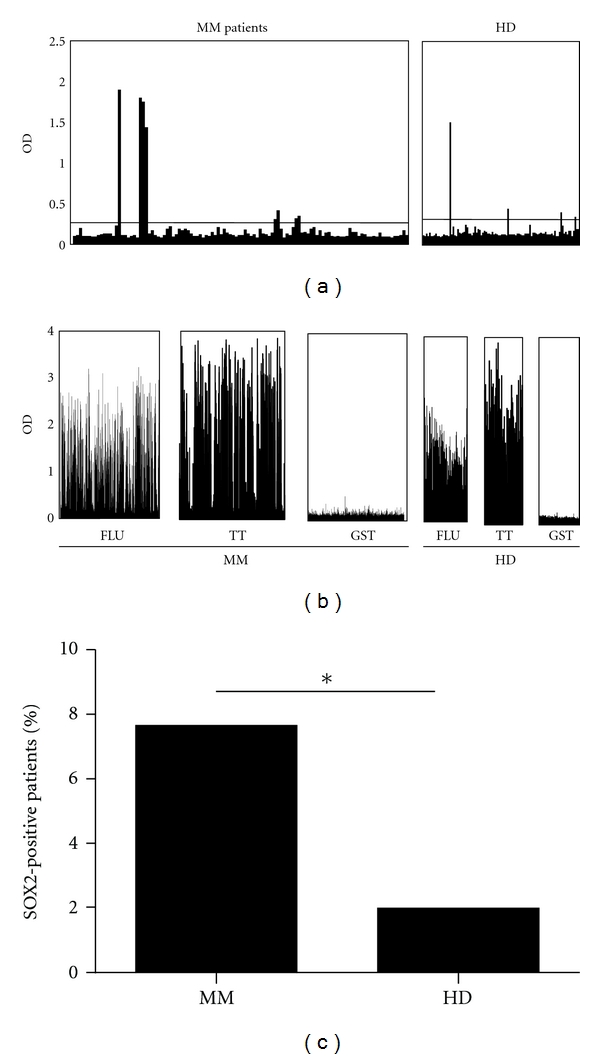
(a) Analysis of SOX2-specific IgG antibody responses in MM patients (*n* = 1094) and in healthy donors (*n* = 100). Results are shown as optical density (OD) at 405 nm. Horizontal bar represents the cut-off value for positivity (OD > 0.274). (b) Analysis of influenza-nucleoprotein- (FLU-), tetanus-toxoid- (TT-) and glutathione-S-transferase- (GST-) specific antibody responses in the same collective of MM patients and healthy donors. (c) Incidence of SOX2-specific antibody responses in the group of MM patients compared with the group of healthy donors (7.7% versus 2.0%). We found significantly more individuals with SOX2-specific antibody responses in the MM group than in the healthy donor group (*P* < 0.05).

We next screened a large number of sera (*n* = 1094) consecutively collected from myeloma patients (*n* = 196) as well as sera from healthy blood donors (*n* = 100) for antibody responses against SOX2. Analyzing a median number of 5.4 (range 1–47) serum samples collected per patient during a median follow-up period of 11.4 months (range 1–39 months), we found 7.7% (15/196) of MM patients and 2% (2/100) of healthy donors to experience autoantibodies against SOX2 (Figures 2(a) and 2(b)). Out of all samples consecutively collected from our myeloma patients, 2% (68/1094) were positive for anti-SOX2 IgG antibodies. Overall, myeloma patients showed a significantly higher frequency of anti-SOX2 antibodies than healthy controls (*P* < 0.05, Figure 2(c)).

To address the general seroreactivity of the included patients and donors against common microbial and viral antigens, we screened all samples for the presence of antibodies against influenza virus nucleoprotein (FLU) and tetanus toxoid (TT). Importantly we did not detect any difference in the frequency of naturally occurring or vaccine-induced antibody responses between myeloma patients and healthy donors (Figure 2(b)). This result strongly suggests that frequency of anti-SOX2 immune responses in MM patients was not influenced by a general hypogammaglobulinemia or B-cell hyporeactivity. 

**Figure 3:**
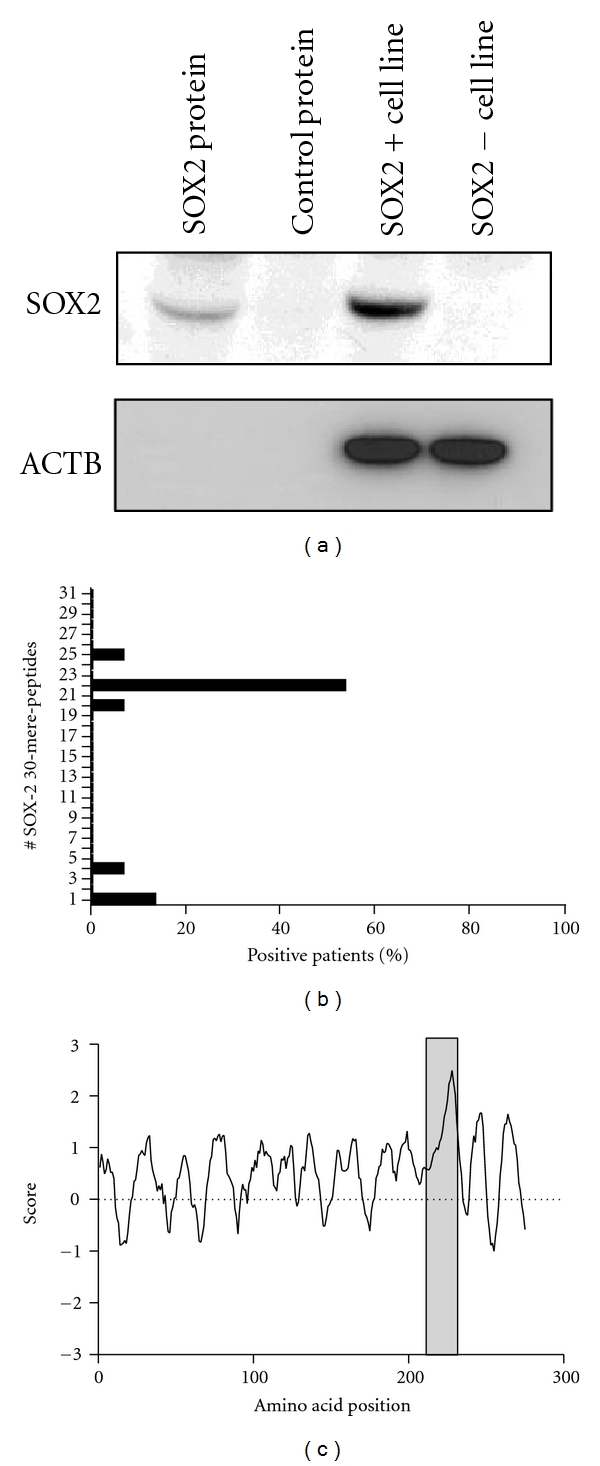
(a) Analysis of specificity of the SOX2 targeted IgG antibody response. Serum from a patient found positive by ELISA was used for western blot analysis and specifically recognized recombinant SOX2 and SOX2 from a SOX2-positive cell line (U266). In contrast, GST and a SOX2-negative cell line (DLD1) remained unstained. ACTB was used as loading control. (b) Mapping of the epitopes of target of the SOX2-specific antibody response in MM patients. Overlapping 20 mer peptides (*n* = 31) spanning the complete SOX2 sequence were used. Percentages of SOX2-antibody-positive patients for each epitope are given on the *x*-axis. Three patients recognized two or three epitopes. 8 patients had SOX2-specific antibodies only directed against the 20 mer 22 (amino acids 211–230), and the antibodies of five patients did not recognize any of the 20 mers that were used. (c) Epitope prediction of the antibody response for the whole SOX2 protein sequence using a hidden Markov prediction model. For each region probability scores are calculated. The grey area represents the main 20 mers of target by the SOX2 antibody response in MM patients.

To confirm the specificity of our patients' serum antibodies, recognition of SOX2 was analyzed by western blot (Figure 3(a)). We found that the IgG antibodies in the patients' sera recognized both the recombinant SOX2 protein and SOX2 protein expressed by a myeloma cell line (U266) shown to be positive for SOX2 (Figure 3(a)). On the other hand, a control protein and a SOX2-negative tumor cell line (DLD-1) remained undetected by the patient serum.

### 3.3. SOX2_211–230_ Represents an Immunodominant Epitope Recognized by Autoantibodies in Myeloma Patients

To further address the specific target of the anti-SOX2 antibody responses, we mapped epitopes recognized using 31 overlapping 20 mer peptides spanning the complete sequence of the antigen. In one-third (5/15) of the patients we were not able to detect any peptide-specific responses suggesting that the respective antibodies might recognize conformational epitopes. However, in the majority of the seropositive patients (53.3%) SOX2-specific antibodies targeted amino acid region 211–230 (Figure 3(b)). Other epitopes were much less frequently recognized by the patient-derived anti-SOX2 IgG antibodies. Using a hidden Markov prediction algorithm we predicted the potential epitopes of target of a SOX2 specific-antibody response (Figure 3(c)). Remarkably, the region with the highest score was indeed the region preferentially targeted by the majority of SOX2-specific antibody responses (211–230).

### 3.4. SOX2-Specific Autoantibodies Are Preferentially Induced after Allogeneic Stem Cell Transplantation

**Figure 4:**
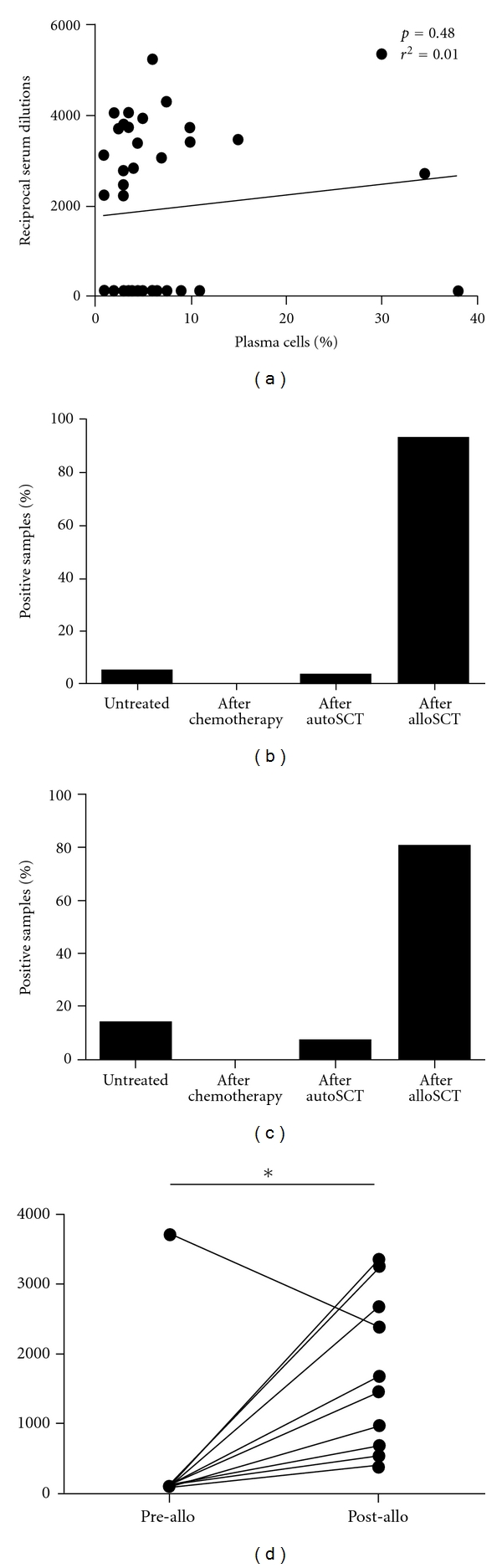
(a) Correlation analysis between the percentage of plasma cells found in the BM of MM patients with the corresponding SOX2-specific antibody titers. For some patients several samples from different time points were analyzed. No significant correlation was found between these two parameters (*P* = 0.4826, *r* = 0.01153). (b) Treatment-dependent distribution of SOX2-positive samples and SOX2-positive patients. 92% (63 of 68) of all SOX2-antibody-positive samples were collected after alloSCT, while 4% (3 of 68) and 2.9% (2 of 68) were collected at time of diagnosis or after chemotherapy, respectively. 80% of SOX2-antibody-positive patients had received alloSCT as maximum treatment, while 13% (2 of 15) and 7% (1 of 15) were untreated or had received autoSCT, respectively. (c) Comparison between SOX2-specific antibody titers before and after alloSCT. Mean values of titers for the respective patient prior and after alloSCT are shown. From 12 SOX2-antibody-positive patients, pre-alloSCT samples were available for 10 patients. 9 of those patients were antibody negative prior to alloSCT and subsequently seroconverted. SOX2-specific antibody titers were significantly higher after alloSCT when compared to pre-alloSCT titers (*P* < 0.05).

In order to understand which clinical factors might be associated with the development of anti-SOX2 antibodies in MM, we next correlated a number of clinicopathological attributes of our patients with the presence or absence of such serological responses. As expected from our observation of a missing association between SOX2 antigen expression and the number of BM-infiltrating plasma cells, we did not observe a correlation between the presence of anti-SOX2 antibodies and the tumor load in the respective patient (Figure 4(a)). Most of the remaining clinicopathological parameters also lacked an association with the appearance of a humoral response against SOX2 (Table 1).

Since myeloma treatment, particularly stem cell transplantation, has immune-modulating properties, we finally investigated the relationship between therapeutic interventions and the occurrence of SOX2-specific antibodies in the myeloma patients. Remarkably, 92.6% (63/68) of all samples found positive for anti-SOX2 antibodies were collected after the patient had received alloSCT. In contrast, only 4.4% (3/68), 0% (0/68), and 2.9% (2/68) of the SOX2 antibody-positive samples were derived from time points when the patient had been treatment naïve, had only received conventional chemotherapy, or had been treated with autologous stem cell transplantation (autoSCT) as maximum therapy (Figure 4(b)). Accordingly, 80.0% (12/15) of the anti-SOX2 antibody-positive MM patients had received alloSCT as maximum therapy while only 13.3% (2/15), 0% (0/15) and 6.6% (1/15), were treatment naïve or had been treated with conventional chemotherapy or autoSCT as maximal therapy, respectively (Figure 4(c)). Importantly, anti-SOX2 antibodies are not likely to be a marker of an unspecific graft-versus-host reaction, since none of the SOX2 antibody positive patients suffered from GvHD at any time point after alloSCT.

To further address the impact of alloSCT on SOX2-specific immunity, we screened samples from the alloSCT, patients taken before transplantation. From 10 available pre-alloSCT samples 9 were found to be negative for SOX2 antibodies prior to alloSCT and these patients had experienced a seroconversion at a median of 21.5 months (range 1–87 months) after transplantation (Figure 4(d)). This observation strongly suggests that immunological mechanisms induced by alloSCT may be capable of breaking tolerance towards SOX2.

To investigate if, as suggested before, SOX2 antibodies may protect from disease progression or recurrence [6], we correlated SOX2 antibodies with the clinical remission status of the patient. We found seropositive samples to be evenly distributed between patients with clinical remission (56%) or progressive disease (44%), respectively (see Supplementary Figure 1 in Supplementary Material available online at doi:10.1155/2011/302145). This finding suggests that there is no immediate connection between the presence of anti-SOX2 humoral immunity and the clinical response of a given MM patient.

## 4. Discussion

We hereby report SOX2 to be expressed in all tissues we analyzed (Figure 1(a)), which is in contrast to previous studies where SOX2 expression has been reported to be restricted to certain tissues (among others neural, stem cell, or tumor tissue) [3, 18]. However, the EMBL-EBI database (http://www.ebi.ac.uk/gxa/gene?gid=P48431) which comprises a meta-analysis of all gene expression data available for SOX2 reports expression in all parts of the human body and in many disease states. In particular, this database reports an expression of SOX2 in plasma cells and myeloma cells, as confirmed by our data (Figure 1). In our current study, SOX2 was not differentially expressed in the BM of healthy donors when compared to the BM of MM patients, an observation which would be in line with the latter analysis. While plasma cells in general might indeed express comparably high levels of SOX2 [19, 20], we believe that SOX2 is by no means tumor or myeloma specific. This assumption is further supported by our flow cytometry data (Figure 1(b)) which suggests a similar expression of SOX2 in the BM of MM patients, in MM cell lines, and in the BM and PB of healthy donors. 

A single study has previously addressed SOX2-specific antibodies in plasma cell disorders [6]. Spisek and colleagues reported SOX2-specific antibodies in 23% of MGUS patients and in none of the MM patients screened, which is in opposition to our results. Here, we have shown that 7.7% of the tested MM patients experience SOX2-specific humoral immunity. This discrepancy may have at least two different reasons. First, the comparably low number of MM patients included (49 versus 196) may have limited the power of the previous study to detect antibody-positive subjects. Second, we describe herein an association between the application of alloSCT and the development of anti-SOX humoral responses. However, none of the patients described in the previous study had received alloSCT, and, therefore, patients with established MM developing anti-SOX2 antibody responses might have simply been missed.

To the best of our knowledge this is the first study characterizing target epitopes of anti-SOX2 antibody responses. We identified a region of the SOX2 protein which was targeted by the IgG antibodies of the vast majority of seropositive patients. Interestingly, our experimental results were in line with the predictions of an online algorithm [13] naming regions potentially recognized by anti-SOX2 antibody responses. Both approaches described SOX2_211–230_ as an immunodominant epitope. This finding might help to improve SOX2-related immunomonitoring techniques in MM or other diseases such as lung cancer but may also be of use for the design of future immunotherapies targeting SOX2.

AlloSCT induces complex processes in the recipient during which autoantigens and/or tumor-associated antigens may become immunogenic [21, 22]. It is well known that immune responses, in particular T-cell responses, appearing after alloSCT mediate the fatal graft-versus-host disease (GvHD) [23]. On the other hand, donor-derived tumor-specific immune reactions induced by transplantation are central for the therapeutic potential of alloSCT [24]. Transplantation-induced immunity, in the framework of a graft-versus-tumor effect, is capable of attacking malignant cells. Accordingly, we and others have shown that alloSCT induces immune responses against tumor-specific or overexpressed antigens [12]. In the case of the SOX2 antigen 80% of the seropositive patients had been treated with alloSCT, and most of these patients had been antibody negative prior to transplantation. Thus, we consider it likely that alloSCT might indeed induce SOX2-specific immunity.

What is the biological meaning of the alloSCT-induced SOX-specific immunity? First, anti-SOX2 immune responses might indeed have an (positive or negative) effect on tumor progression. At this time we have no evidence supporting this hypothesis since the occurrence of anti-SOX2 antibodies in our myeloma patients was not related to the disease burden or the remission status. Such an observation would be in line with studies on patients with lung cancer which also failed to detect any association of SOX-specific antibodies with the prognosis of the patients [5, 25, 26].

On the other hand, anti-SOX2 immunity (maybe in concert with immune responses against a multitude of other autoantigens) might simply be a sign of autoimmune or even alloimmune disease, that is, occurring in the framework of a GvHD reaction. In our current study, we did not detect an association between the occurrence of GvHD and the presence of anti-SOX2 antibodies. In fact none of the SOX2-antibody-positive patients who had been treated with alloSCT experienced acute or chronic GVHD.

Since on the one hand SOX2-specific immunity does not seem to be associated with a more favorable course of the disease and on the other hand it does not correlate with a graft-versus-host reaction, the biological meaning of such an immune response remains unclear. We will need to perform prospective studies in MM in cohorts well balanced for treatment and for stage of the disease to understand this immune reaction. Eventually, knowledge gained through such studies will help us to decide whether SOX2 represents a promising prognostic or therapeutic target for patients with multiple myeloma.

## Figures and Tables

**Table 1:** Patient characteristics. Data are shown for all patients and for the subgroup of SOX2-seropositive patients. LC: light chain; HC: heavy chain.

Parameter	Total	SOX2 seropositive	Significance
Sex			n.s.
Male	115	9	
Female	80	6	

Age			n.s.
>60	69	6	
≤60	126	9	

Karyotype*			n.s.
Normal	83	7	
Complex	15	0	
del13q14	46	6*	
del17p13	12	3	
*t*(4;14)	9	0	
Not tested	30	0	

LC isotype			n.s.
Light lambda	62	6	
Light kappa	100	7	

HC isotype			n.s.
IgG	167	13	
IgA	18	0	

Stage			n.s.
I	32	2	
II	52	2	
III	95	9	

*One patient was found to bear a 13q14 and a 17p13 deletion.
